# Highways and Detours in the Realm of Photodynamic Therapy

**DOI:** 10.3390/ijms25063119

**Published:** 2024-03-08

**Authors:** David Kessel, Qian Peng

**Affiliations:** 1 Department of Pharmacology, Wayne State University School of Medicine, 540 E. Canfield Street, Detroit, MI 48201, USA; 2 Department of Pathology, Oslo University Hospital, N-0310 Oslo, Norway; qian.peng@rr-research.no; 3 Department of Optical Science and Engineering, School of Information Science and Technology, Fudan University, Shanghai 200433, China

**Keywords:** photodynamic, photosensitization, cancer

## Abstract

Photodynamic therapy (PDT) has been a topic of interest since the first report in 1900 but has yet to become a ‘mainstream’ treatment protocol in the medical field. There are clear indications for which PDT might be the ‘method of choice’, but it is unlikely that there will be protocols for the treatment of systemic disease. This report discusses recent developments for promoting PDT efficacy, in the context of what is already known. Factors that can limit the scope of these applications are also indicated. Among the more interesting of these developments is the use of formulation techniques to target specific organelles for photodamage. This can enhance responses to PDT and circumvent situations where an impaired death pathway interferes with PDT efficacy.

## 1. Introduction

The concept of sensitizing organisms to light has been known since prehistoric times when photobleaching of material by sunlight was practiced, but the first scientific publication on the topic appeared in 1904 [1]. The dye eosin was found to sensitize microorganisms to external irradiation, resulting in loss of viability. Numerous reviews on the topic of what has come to be known as ‘photodynamic therapy (PDT)’ have since appeared; a few will be cited and discussed here [2,3,4].

The three essential components of PDT are [a] a photosensitizing agent, [b] light at a wavelength that corresponds to an absorbance optimum of the photosensitizer, and [c] molecular oxygen. In order for them to be a selective effect, it is necessary for the photosensitizer to become concentrated in target cells, e.g., sites of neoplasia, compared with adjacent normal cell types. It is common to find photosensitizing agents also being accumulated by several host organs, e.g., liver, spleen, and kidney [5]. Such sites are normally protected from light and are thereby spared from adverse effects. Since this is a common observation, protocols where the photosensitizer formulation carries a light source will likely be non-selective, with the capacity to produce possibly substantial adverse effects in tissues that tend to accumulate photosensitizing agents.

Many photosensitizers have been described in the literature. Among the important factors predicting clinical success are absorbance profiles and the ability of excited states to efficiently transfer energy to oxygen, creating reactive oxygen species (ROS). The latter are responsible for the observed cytotoxic effect. It has been established that only the longer wavelengths of light can penetrate deeper into tissues. This can readily be demonstrated by looking towards sunlight with eyes closed. The red glow that appears is the result of the ability of the thickness of the eyelid to screen out shorter wavelengths of light. The presence of infrared cannot be detected by the receptors in the retina but is responsible for the heating effects of direct sunlight.

Death pathways associated with PDT involve three separate effects. One is termed ‘direct photokilling’, which will be discussed in greater detail. Other effects, observed only in vivo, include a selective shut-down of the tumor vasculature and the eliciting of an immune response to photodamaged cells. The latter effect has the capacity to affect malignant cells outside of the field of irradiation. These topics are covered amply in the review articles cited above. It is important to realize that in vitro studies can only provide information on effects related to direct photokilling. Moreover, monolayers of photosensitized cells can readily be eradicated by shorter wavelengths that will not penetrate more than a few cell diameters. An additional concern in the translation of in vitro studies to the clinical situation is that in vitro studies cannot reliably distinguish photosensitizers with no special affinity for sites of neoplasia from those that might be useful for selective photokilling.

## 2. The Relevance of Preclinical Studies

Measurement of cell viability after photodamage in vitro involves a variety of procedures, as a function of the protocol being examined. The most direct and unambiguous involves animal models where effects on the growth of implanted tumors can be observed. The expense of such studies usually precludes the use of this protocol for the initial evaluation of efficacy. It is, however, true that no protocol would be approved for clinical use unless efficacy is shown in animal models. These usually involve tumors implanted subcutaneously or, less often, in orthotopic models. The latter means that, e.g., a glioma will be implanted in the brain, a hepatoma in the liver, etc. It is more common to use subcutaneous implants. While in vitro studies can provide an indication of selectivity, orthotopic models may more reliably predict clinical efficacy. Studies involving subcutaneous tumors can provide an indication of the probability of clinical success. Where host sites might be expected to accumulate photosensitizers, e.g., liver, orthotopic studies using liver tumors will be needed to indicate the likelihood of success for a proposed protocol involving hepatomas.

There are many examples in the PDT literature where efficacy is investigated by studies in cell culture involving exposure of malignant cell types to photosensitizers and light. These usually involve tumor monolayers but can also utilize ‘3D’ systems where tumor aggregates are grown in appropriate media, usually ‘soft agar’. While the latter approach may provide more accurate estimates of what might occur in vivo, the expense and difficulty with assessing results can limit such studies. With tumor monolayers, it is relatively simple to carry out clonogenic assays where growth after PDT is monitored by colony counting. While this can be an expensive and time-consuming approach, it will unambiguously report on the ability of a given protocol to eradicate malignant cells.

There are several issues to be remembered when attempting to predict the efficacy of a given protocol from monolayer culture studies. Such systems are readily treated with light of any wavelength, including shorter wavelengths that will be scattered in vivo. The use of light at wavelengths of 630 nm (and preferably farther into the red and near IR) is necessary for them to be a significant depth of photokilling by light. There is a requirement that cells have a high cloning efficiency, i.e., readily produce colonies from single cells. Moreover, in vitro studies generally involve the use of a single cell type. Spontaneous tumors tend to be heterogeneous. While efficacy in a monolayer model may be encouraging, it is important to remember that the results may represent an idealized system and will not necessarily predict clinical success. Many spontaneously arising malignant cell types have unusual growth requirements and can be difficult to adapt to cell culture without a selection process that may alter photodynamic responses.

In vitro studies can identify agents that catalyze photokilling and provide an indication of relevant death pathways. Since many photosensitizers fluoresce when excited at an appropriate wavelength, it is usually feasible to use fluorescence microscopy for the identification of sub-cellular site(s) of photosensitizer localization. This involves a comparison of patterns of sensitizer fluorescence with those of known fluorescent markers for mitochondria, ER, lysosomes, lysosomes, and other organelles. Prior studies have indicated that mitochondrial photodamage generally leads to the release of cytochrome c into the cytoplasm, a trigger for apoptosis [6].

Other death pathways can be triggered by lysosomal and endoplasmic reticulum (ER) photodamage; damage to many sub-cellular sites has been shown to promote and/or amplify photokilling. One example involves targeting both lysosomes and mitochondria. This approach can significantly amplify the apoptotic response: lysosomal photodamage releases factors that can promote photokilling by apoptosis [7].

When malignant cell types have an impaired response to apoptotic stimuli, photokilling can be accomplished by targeting the ER [8]. This has been shown to lead to a death pathway termed ‘paraptosis’. This route ultimately results in cells filling with cytosolic vacuoles and eventually losing their cytoplasm, leaving behind bare nuclei that are unable to proliferate [9]. Paraptosis can be an effective means for the eradication of cell types that fail to show an apoptotic response [8]. Studies on sub-cellular targeting as a function of PDT efficacy are becoming more common and represent an example of the relevance of research efforts designed to identify new protocols that will be useful if the apoptotic pathway to death is impaired.

## 3. Determinants of Efficacy from In Vitro PDT Studies

One of the more interesting lines of research involves the promotion of photokilling by enhancing the apoptotic response to PDT [7]. Lysosomal photodamage was found to result in the release of calcium ions from damaged lysosomes. This can activate the enzyme calpain which cleaves the protein termed autophage-related gene 5 (ATG5) to a fragment that can promote apoptosis. Formulations of the photosensitizer termed BPD can be designed that target both lysosomes and mitochondria. The result is an enhanced apoptotic effect that substantially promotes PDT efficacy. This result appears to explain a 1996 report that indicated a substantial promotion of PDT efficacy for the treatment of relatively large tumors in the rat [10]. The protocol involved two photosensitizers administered together but activated sequentially. This was possible since each had a different absorbance profile and a different localization pattern. The optimal effect was produced when lysosomal photodamage occurred before mitochondrial photodamage. This can be explained by the short half-life of the ATG5 fragment. Lysosomal photodamage must come first in order for the ATG5 fragment to sensitize mitochondria to subsequent photodamage.

Another procedure for promoting PDT efficacy, mentioned in the preceding section, involves targeting the ER. This promoted photokilling in the cell culture of a cell line with an impaired apoptotic response to PDT. The ability of formulation procedures to alter targets for photodamage is mentioned in Ref. [7]. As newer methods are developed that can target specific organelles for photodamage, it can be feasible to fine-tune photodamage for optimal efficacy.

Many investigators have taken advantage of the ability of in vitro studies to identify photosensitizing agents capable of catalyzing photokilling upon irradiation and to acquire information on sub-cellular sites of photosensitizer localization and death pathways. None of these studies will, however, provide any information on in vivo pharmacokinetics, i.e., the persistence of an agent in the circulation and different cells and tissues. In vitro studies also tend not to provide any information on selective photosensitization of malignant vs. normal host cell types or the potential role of vascular shutdown and immunologic effects. Even animal studies, usually involving tumor-bearing mice, cannot always predict clinical efficacy.

An explanation for the selective biodistribution of photosensitizers into malignant cell types has yet to be unambiguously provided. It has been noted that malignant cells tend to exhibit overexpression of lipoprotein receptors. This can lead to reduced levels of plasma lipoproteins [11,12,13]. Photosensitizers that bind to specific classes of lipoproteins may thereby be selectively accumulated in certain cell types. In this regard, it is important to recall that the mouse tends to have high levels of high-density lipoprotein (HDL) in circulating blood, while in humans, the predominant species is low-density lipoprotein (LDL). The mouse may therefore not be an entirely appropriate model for assessing clinical efficacy of PDT protocols.

While the affinity of photosensitizers for circulating lipoproteins may be one factor in selective distribution to sites of neoplasia, an explanation for observed patterns of photosensitizer biodistribution has yet to be devised. With regard to death mechanisms, photokilling was first described in 1904 [1], but a description of a pertinent death pathway was not provided until 87 years later [6]. Prior reports on PDT involved what might be termed ‘phenomenology’. PDT can lead to cell death but the process is a mystery. Photosensitizing agents (usually porphyrins or porphyrin analogs) could be shown to successfully treat tumor-bearing mice bearing subcutaneous tumors or patients with neoplasia. Irradiation was provided by a variety of sources including arc lamps, with the laser a relatively recent development. Success was evaluated in terms of decreased tumor size as a function of time after irradiation. Vascular shut-down was also described [14,15,16] several years before Oleinick’s observations on apoptosis, but it was clear that this effect could not explain photokilling in cell culture [6].

While there was no comprehensive explanation for photokilling prior to 1991, the nature of the commonly used agent HPD (hematoporphyrin derivative) was also a mystery. The structure of HPD had been determined a year earlier. The name ‘hematoporphyrin derivative’ was used because nobody knew what it was. HPD had been prepared by Schwartz and Lipson who only knew that their procedure produced a tumor-localizing agent that was from hematoporphyrin. The process consisted of treating hematoporphyrin with a mixture of acetic and sulfuric acids, followed by neutralization with sodium hydroxide [2]. The rationale for the use of this procedure was never fully explained. In 1990, Pandey’s group at the Roswell Park Cancer Center in Buffalo determined that HPD consisted of a collection of monomers (hematoporphyrin and other reduced porphyrins), along with ether-linked dimers, trimers, and higher oligomers of these structures. The dimers and oligomers were mainly responsible for the anti-tumor efficacy of HPD [17].

An agent currently in clinical use, Photofrin, is a somewhat purified version of HPD, lacking the monomers (hematoporphyrin, protoporphyrin, and an intermediate porphyrin, bearing only one vinyl group). The principal components are porphyrin dimers and higher oligomers. This agent is still widely used although it tends to localize in skin along with tumors, resulting in a somewhat lengthy interval of skin photosensitization. Better agents were gradually developed. The details are amply discussed in reviews, e.g., in Ref. [3], and will not be considered here. Many studies were directed at assessing the biodistribution of HPD and other photosensitizers as they were identified. Most photosensitizers appear to be predominantly accumulated by the liver. This organ is normally spared from adverse phototoxic effects by being spared from irradiation.

## 4. PDT: What Can Go Wrong

It is not feasible to test every new and potentially interesting photosensitizer in the clinic. The regulatory agencies require proof of safety and efficacy in animal models before approval will be granted for clinical studies. As explained above, the mouse, while an inexpensive small animal, may not be the ideal predictive model for clinical success. Using strictly in vitro procedures to identify suitable agents requires a bit of caution.

When treating monolayers of cells, any wavelength can be suitable for eliciting a photodynamic effect, but the use of wavelengths much below 630 nm will limit the depth of light penetration into solid tumors. There have been many studies on the ability of different wavelengths of light to penetrate tissues, with the first having been described by Wilson’s group [18]. If tumors tend to occur in monolayers, e.g., bladder cancer, this may not be an issue. Otherwise, absorbance at longer wavelengths will be required. Other in vitro models have periodically been utilized, including, notably, the use of ‘3D’ cultures involving tumor aggregates [19]. These may more closely correspond to the environment of tumors in vivo and it is feasible to culture different cell types. Results may more closely resemble the in vivo situation, but the format makes it somewhat more difficult to assess the efficacy of a procedure since clonogenic assays cannot easily be carried out.

The presence of oxygen is necessary for PDT efficacy. There have been some studies relating to PDT efficacy in hypoxic environments (reviewed in Ref. [20]). It is not clear whether any such approaches will be successful in clinical studies. Large tumors with central hypoxic regions will likely be poor targets for PDT without prior surgical debulking.

In addition to absorbance spectra, other factors can affect PDT efficacy. It can be difficult to assess selective localization in neoplasia vs. nearby host tissues by examining only malignant cell types in monolayer culture. Sub-cellular sites of localization can be assessed by fluorescence microscopy since most photosensitizing agents show fluorescence upon excitation at an absorbance optimum. Identifying ROS formation requires a suitable protocol. These species have a very short half-life [21] so it is necessary for the detecting agent to be present during irradiation. Otherwise, only hydrogen peroxide and a few other long-lasting species, e.g., lipid peroxides, will be detected.

An unambiguous method for detecting photokilling in vitro involves the use of clonogenic assays to examine the ability of cells to proliferate. The ‘MTT’ assay and assorted variations only assess levels of mitochondrial dehydrogenases. The MTT assay has been shown unreliable for identifying photokilling [22]. This and similar procedures do not necessarily provide an accurate indication of loss of viability. But, many malignant cell types have unusual growth requirements and are not readily cultured. Moreover, spontaneously arising tumors are generally heterogeneous, and their response to PDT may not be predicted from studies involving only cloned cells.

## 5. Photodiagnosis and PDT with 5-Aminolevulinic Acid (5-ALA) and Its Derivatives

**Figure 1:**
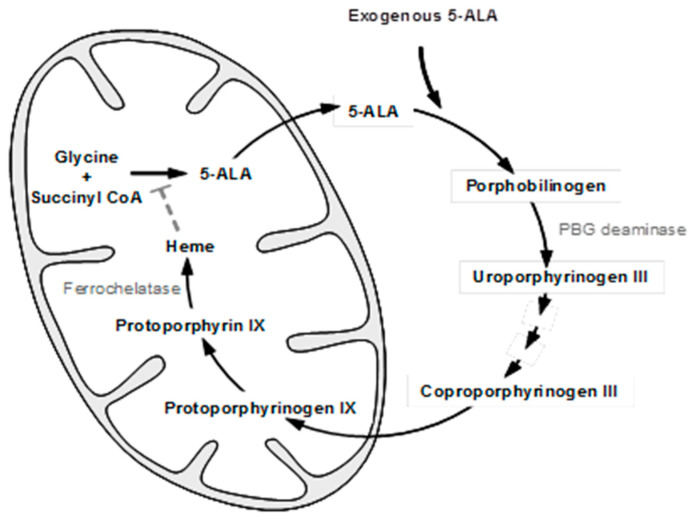
Heme biosynthetic pathway.

As PDT with chemically synthesized photosensitizers has a major side-effect of skin phototoxicity, considerable interest has been directed toward developing a new PDT regimen that relies on an endogenously synthesized photosensitizer [23]. In the first step of the heme biosynthetic pathway (shown in Figure 1), 5-ALA is formed from glycine and succinyl CoA. The last step is the incorporation of iron into protoporphyrin IX (PpIX, a potent photosensitizer), which takes place in the mitochondria under the action of the rate-limiting enzyme, ferrochelatase. By adding exogenous 5-ALA, the naturally occurring PpIX may accumulate because of the limited capacity and/or low activity of ferrochelatase. Porphobilinogen (PBG) deaminase is another enzyme of the heme synthesis pathway. Its activity is higher in hyper-proliferative cells, while that of ferrochelatase is lower, so PpIX accumulates with a high degree of selectivity in these cells [24]. Such selectivity may also be due to the fact that actively dividing cells overuse intracellular iron stores for their cytochrome and DNA syntheses and thus do not convert PpIX into heme efficiently. This intracellular PpIX accumulation has been exploited for its PDT of skin diseases (3), starting from Kennedy, et al. in the early 1990s [23]. Since 5-ALA is a hydrophilic molecule and has a limited ability to penetrate biological barriers, esterified derivatives of 5-ALA with enhanced hydrophobic properties were developed in the mid-1990s [25,26]. Clinically, topical PDT with 5-ALA or its methylester is an established modality for skin premalignant and non-melanoma malignant disorders [27,28,29]. Moreover, photodetection with oral administration of 5-ALA for fluorescence-guided surgical resection of glioma [30] and with intravesical instillation of hexaminolevulinate for bladder cancer [31] are also established modalities today. Recently, 5-ALA is also used for a clinical trial in the extracorporeal photopheresis of patients with graft versus host disease [32]. Such successful clinical applications with PpIX precursors have detoured the mainstream of traditional PDT. Preclinical research on this technology has also included the use of dendritic derivatives of 5-ALA [33] or the conjugation of the dendritic 5-ALA with an iron-chelating agent [34] to enhance intracellular PpIX accumulation for PDT. In addition, Vitamin D and other differentiation-promoting agents have been found to enhance the efficacy of 5-ALA-PDT [35].

## 6. Looking Back

At a 1989 meeting at the Ciba Foundation in London, where almost every major figure in the PDT field provided research updates, there was not one word concerning PDT-mediated death mechanisms [36]. There were many presentations relating to clinical trials and encouraging results and an appreciation of the need for light that could penetrate tissues. It was realized that PDT could interfere with blood flow to tumors in vivo, but no details were presented concerning the mechanism(s) of direct photokilling. In the ensuing years, critical components of the biochemistry and biophysics of the process have been discovered and reported. The incorporation of PDT into clinical practice has, however, been elusive.

## 7. Conclusions and Future Directions

Photodynamic therapy is perhaps best defined as a ‘specialty’ procedure. In its present form, it is not suitable for dealing with systemic diseases, e.g., leukemias or widely disseminated tumors. Since light is required, tumors of unknown origin are not appropriate targets. Where clearly delineated tumors are identified, PDT can be very effective and is relatively free from the adverse reactions associated with chemotherapy or protocols involving ionizing radiation.

Forty years have elapsed since the early work from Dougherty’s group and others initiating what might be termed ‘the PDT era’. After initial speculation that PDT might somehow be the ‘magic bullet’ for cancer treatment, there is a better appreciation of the pertinent limitations and indications. PDT clearly has a niche: when properly applied, it can produce effects with minimal host toxicity. Some of these, along with a historical background, were described in a small volume written by Dr. Dougherty [37]. At present, PDT is available for clinical use at a few specialized centers. This is likely to remain true unless considerable progress is made in simplifying dosimetry and irradiation procedures.

The details concerning the details and application of PDT will necessarily depend on the site(s) of neoplasia. For some indications, shorter wavelengths of light may be adequate, e.g., in the treatment of bladder tumors. Otherwise, longer wavelengths, to which tissues are transparent, will be required to produce a significant level of tumor eradication. Preclinical studies have been helpful in identifying potentially useful photosensitizing agents and delineating determinants of clinical success. These are outlined in the text.

It must be remembered that preliminary in vivo data usually involves small animals, e.g., mice, which will necessarily have correspondingly small tumors that are not spontaneous, having been transplanted by the investigator. Few reports from the literature involve orthotopic implants. The relevance of successful protocols that involve subcutaneous tumors to those that occur in the brain, lung or other organs is therefore not entirely clear. Studies involving orthotopically implanted tumors are seldom encountered in the PDT literature.

The PDT literature also contains many reports involving clinical studies. These will necessarily involve spontaneously arising tumors, which are typically very heterogeneous. As a result, there will likely be cell types present that vary in their response to PDT procedures that may have been successful when applied to a uniform cell population in an animal model. Successful clinical studies are, however, the goal of any PDT research program. The late Sidney Farber, who initiated the first successful treatment for childhood leukemia [38], strongly supported ‘basic research’. But, he was often heard to say that ‘the best way to cure mouse leukemia is not to implant it’. It is generally agreed that research into cancer control and eradication must necessarily involve cell cultures and animal models, but translation to the much more complex clinical situation is seldom simple.

In Table 1, four examples of photosensitizing agents are shown that vary in optimal wavelength values. The relative advantages and disadvantages of each are briefly presented. Photofrin is a well-known agent and there is substantial experience in its use. The major disadvantage is the persistent photosensitization of skin. While the 630 nm wavelength has been shown to be adequate for clinical efficacy, the depth of penetration will be inferior to agents with absorption in the far red or near IR. BPD can be formulated so as to promote PDT efficacy and has a somewhat longer absorption optimum. As indicated in Ref. [3], this agent was initially developed for the treatment of ocular issues. TLD-1433 is a ruthenium-based agent with a limited absorption spectrum [39]. It may be useful where very thin layers of tumor are to be treated, e.g., in the bladder. This property can also be a disadvantage if deeper layers of the tumor are to be treated. ALA and its methylester and hexylester are probably the most widely used in clinical applications of photodiagnosis and PDT today. They are effective for photodetection of glioma and bladder cancer and PDT of superficial lesions with few adverse reactions. It should, however, be pointed out that PDT with ALA or its derivatives is not effective for a thick lesion due to the fact that a negligible amount of PpIX is produced in those hypo-proliferative vascular endothelial cells of the lesions.

These four agents represent typical examples of the collection of agents for which there is broad PDT experience. Critical elements of efficacy include adverse effects, the scope of light penetration, and clinical experience. There are many other determinants of efficacy, but what appears to be the most critical of these is pointed out. It appears that no single photosensitizing agent will be optimal for every potential indication, just as no single agent is indicated for all cases where cancer chemotherapy is indicated.

With regard to future directions, PDT is currently proceeding along a variety of lines, some more appropriate than others. Many reports in the literature involve agents with no significant absorbance in the red or near infrared. These will seldom be useful in a clinical situation aside from very special situations. Often, preclinical efficacy is evaluated by inappropriate methods that do not involve clonogenic or other procedures where cell proliferation is actually measured.

Granting agencies continue to support PDT studies and a few pharmaceutical companies have become interested in the field. It is true that financial support for research proposals generally involves prior approval of a research plan by a study section or other evaluation committee. These groups do not always contain reviewers with substantial experience in PDT research. Early efforts to establish such support had only limited success.

Dougherty often commented on the first NIH study section that reviewed one of his proposals. In the resulting critique was the sentence ‘we all know light does not penetrate tissues’. His group had many adventures in attempting to interest pharmaceutical organizations in PDT, as outlined in Ref. [10], but were ultimately successful in obtaining regulatory approval in the US and elsewhere. It remains to be seen whether PDT will eventually become a standard treatment for localized neoplasia and perhaps localized microbial infections. Whether PDT will ever be useful for systemic disease remains an open question.

## Figures and Tables

**Table 1:** Comparison of properties of representative PDT agents.

Photosensitizer	Optimal Wavelength (nm)	Advantages	Disadvantages
Photofrin	630	regulatory approval established efficacy	persistent skin photosensitization
BPD	690	better tissue penetration formulation feasible	limited regulatory approval
TLD-1433	530	limited tissue penetration	limited tissue penetration
5-ALA and its derivatives	405/630	regulatory approval effective for superficial lesions photodetection no risk of photosensitization	ineffective for thick lesions
